# The Effect of Reduced Learning Ability on Avoidance in Psychopathy: A Computational Approach

**DOI:** 10.3389/fpsyg.2019.02432

**Published:** 2019-11-01

**Authors:** Takeyuki Oba, Kentaro Katahira, Hideki Ohira

**Affiliations:** ^1^Department of Psychology, Graduate School of Environmental Studies, Nagoya University, Nagoya, Japan; ^2^Department of Psychology, Graduate School of Informatics, Nagoya University, Nagoya, Japan

**Keywords:** psychopathy, reinforcement learning model, learning rate, prediction error, avoidance learning

## Abstract

Individuals with psychopathy often show deficits in learning, which often have negative consequences. Several theories have been proposed to explain psychopathic behaviors, but the learning mechanisms in psychopathy are still unclear. To clarify the learning anomalies in psychopathy, we fitted reinforcement learning (RL) models to behavioral data. We conducted two experiments to examine the effect of psychopathy as a group difference (Experiment 1) and as a continuum (Experiment 2). Forty-three undergraduates (in Experiment 1) and fifty-five undergraduate and graduate students (in Experiment 2) performed a go/no-go based learning task with accompanying rewards or punishments. Although we observed no differences in learning performance among the levels of psychopathic traits, the learning rate for the positive prediction error in the loss domain was lower for those with high-psychopathic trait than for those with low-psychopathic trait. This finding indicates that individuals with high-psychopathic traits update an action value less when they avoid a negative outcome. Our model can represent previous theories under a computational framework and provide a new perspective on impaired learning in psychopathy.

## Introduction

Psychopathy is a group of personality traits described by callousness, lack of empathy, shallow affect, and impulsivity (Cleckley, 1976), and these traits can be divided into emotional detachment and externalizing behavior (Hare, 2003). Because of such features, individuals with psychopathy often commit antisocial behaviors and harm others (Hemphill et al., 1998; Leistico et al., 2008). However, a wide range of people who do not commit crimes may possess psychopathic traits (Levenson et al., 1995; Gao and Raine, 2010) because impaired emotional functions, rather than impulsivity, constitute the core element of psychopathy (Hare, 2003; Blair, 2006). Indeed, persons with high psychopathy who are recruited from a non-clinical population often show some behaviors similar to those of psychopathic offenders (Lynam et al., 1999; Osumi et al., 2007b; Kahane et al., 2015; Pletti et al., 2017).

One of the remarkable features related to psychopathy is a failure to learn from negative consequences, such as an electric shock, a monetary loss, or a loss of points (Lykken, 1957; Blair et al., 2006; von Borries et al., 2010). Many studies have reported that individuals with psychopathy showed deficient performance in several types of learning that are needed to change one’s own behavior through unpleasant experiences. A major paradigm for the evaluation of learning abilities in psychopathy is a go/no-go based learning task. Individuals with psychopathy often fail to withdraw a response to a stimulus that leads to punishment, but they rarely fail to respond to a stimulus that leads to a reward (Newman and Kosson, 1986; Newman and Schmitt, 1998; Lynam et al., 1999; Finger et al., 2011). Moreover, learning deficits have often been observed among psychopathic persons with low trait anxiety (Lykken, 1957; Newman and Schmitt, 1998). This finding indicates that individuals with psychopathy have difficulty in learning to adjust their behaviors based on negative outcomes. Clarifying the mechanisms of learning with negative results for individuals with psychopathy is thought to be important because learning deficits may cause abnormal moral development and behavior (Blair, 2017).

The reasons why individuals with psychopathy have difficulty learning from negative results have been debated. A classic explanation for the characteristics of psychopathy is the low-fear hypothesis, which suggests that diminished reactions to threatening stimuli underlie psychopathic features (Lykken, 1957; Patrick et al., 1993; Hoppenbrouwers et al., 2016). In this hypothesis, individuals with high psychopathy are less susceptible to negative stimuli; thus, their learning performance is insufficient compared to that of individuals with low psychopathy. In this regard, researchers have developed several neurocognitive models for psychopathy, such as the integrated emotion system (IES) theory, which highlights the amygdala and orbitofrontal cortex (OFC) functions that are assumed to form stimulus-outcome associations and to select appropriate actions after a reversal of contingency (Blair, 2006). In contrast, Newman and colleagues argued that impairments related to psychopathy stem from abnormal attentional systems (Hiatt et al., 2004; Zeier et al., 2009; Newman et al., 2010; Newman and Baskin-Sommers, 2016). This theory, the response modulation hypothesis, assumes that learning impairments in psychopathy occur due to the disregard for a disadvantageous sign while attending to a goal-related stimulus. While these theories have led to important findings, they seem to lack evidence to directly describe the learning deficits.

Reinforcement learning (RL) models can provide insight into the learning deficits in psychopathy by providing a computational framework for describing how advantages are maximized and disadvantages are minimized through experience (Sutton and Barto, 1998). A key component of RL models, especially a delta learning rule, is the prediction error (PE), which is the difference between an anticipated value and an actual received value. Several studies have shown neural activities correlated with the PE algorithms in classical and instrumental learning (Schultz et al., 1997; O’Doherty et al., 2003). This method allows us to summarize large dynamic data sets (i.e., trial-by-trial choice data) with very few parameters, such as a learning rate (i.e., the extent of modification to the error) and a subjective impact of outcomes (i.e., choice randomness). Using the learning parameters, the RL models can map psychopathology, such as schizophrenia (Culbreth et al., 2016) and major depression (Kunisato et al., 2012; Huys et al., 2013). This approach to studying mental illness using computational models is called computational psychiatry (Montague et al., 2012; Huys et al., 2016), and the RL models can provide details regarding learning mechanisms and anomalies. Thus, the RL models can describe learning impairment in psychopathy and explore how it corresponds to the abovementioned theories.

Several pioneering studies have explored the computational characteristics of learning abilities for individuals with psychopathy. Using a reward learning task in which a partner gives advice on the choice of behavior, Brazil et al. (2013a) found that some psychopathic traits were negatively correlated with the weights of the subjective probabilities for reward and social information. Blair et al. (2004) applied a Hebbian learning rule to simulate actual learning performance in psychopathic offenders and revealed that a model that represented impairments in stimulus-punishment associations could replicate the performance of individuals with psychopathy. Aisbitt and Murphy (2016) identified a learning characteristic related to psychopathy from a learning model thought to be affected by attention. They showed that the effect of competing cues in a learning task decreased with the extent of psychopathy; this result was predicted by the model that Aisbitt and Murphy used. In the go/no-go based learning task, White et al. (2013) demonstrated that adolescents with conduct problems showed smaller blood oxygen level-dependent (BOLD) signals correlated with action values that were estimated from an RL model. Brazil et al. (2017) used a computational framework to model fluctuations of BOLD signals during threat conditioning and showed that psychopathic traits were positively related to the fluctuations. These findings contribute to the theoretical, behavioral, and neurobiological understanding of learning deficits in psychopathy. However, Brazil et al. (2013a) did not test the effect of negative consequences on learning. Blair et al. (2004) and Aisbitt’s studies did not report group differences for the model parameters because these studies used models to predict learning performance. Brazil et al. (2017) and White et al. (2013) mainly examined neural activities related to learning models. Thus, these studies have not examined the learning parameters associated with avoidance learning in psychopathy.

This article aims to examine the learning mechanisms in psychopathy using RL models. These models can provide parameters that characterize certain aspects of learning, and we searched for the relationships between RL parameters and psychopathy. We conducted two experiments to examine the relations of psychopathy as a group difference and as a continuum. We hypothesized that the abnormal learning process in psychopathy is related to aberrant valence systems such as reward-punishment and/or positive-negative PE processes. In line with the low-fear hypothesis, individuals with psychopathy showed poor reactions to fear conditioning (Birbaumer et al., 2005) and weak physiological responses to unpleasant images (Blair et al., 1997; Osumi et al., 2007b). Moreover, the IES theory predicts that psychopathic traits are related to a weaker ability to build a stimulus-outcome association (Blair, 2006). Therefore, according to the low-fear hypothesis and the IES theory, individuals with high psychopathic trait are slow to build negative associations (i.e., having a lower learning rate in a loss domain) than are individuals in a control group. In contrast, the response modulation hypothesis relies on data suggesting that when individuals with psychopathy concentrate on a target stimulus, they tend to attenuate the interference by another stimulus (Hiatt et al., 2004; Zeier et al., 2009). If the response modulation hypothesis is valid, learning parameters related to a reward system (especially the learning rate for positive PE in a gain condition) for the high psychopathic level group were expected to be higher than parameters related to a punishment system. In addition, we sought other parameters that may contribute to learning in psychopathy.

## Experiment 1

We first used an extreme groups approach to compare the effect of the difference between high and low levels of psychopathy on learning parameters. This method has the advantage of increased statistical power (Preacher et al., 2005; Katahira and Yamashita, 2017). The goal of Experiment 1 was to identify which learning parameters were differed among individuals with high and low levels of psychopathic traits.

### Materials and Methods

#### Participants

Data were obtained from 46 undergraduate students who met specific criteria, which are described later. All participants completed the Japanese version of the Levenson Self-Report Psychopathy Scale (LSRP: Levenson et al., 1995; Sugiura and Sato, 2005) and the trait anxiety scale from the Japanese version of the State-Trait Anxiety Inventory (STAI: Spielberger et al., 1970; Shimizu and Imae, 1981). We determined the sample size following previous studies (Brazil et al., 2013a; Aisbitt and Murphy, 2016; Pletti et al., 2017). The participants were divided into high- and low-psychopathic trait groups based on the criteria, and each group consisted of 23 participants. Two participants in the high-psychopathic trait group and one participant in the low-psychopathic trait group were excluded from the analysis because they performed poorly due to misunderstanding the instructions for executing the task in this experiment. Therefore, the students with high-psychopathic trait consisted of 21 participants (15 males and 6 females, mean age = 19.24, *SD* = 0.77), and the students with low-psychopathic trait consisted of 22 participants (13 males and 9 females, mean age = 19.05, *SD* = 0.90). All participants gave their written informed consent and received ¥1,000 for participation. This study was approved by the Ethics Committee of Nagoya University.

When we recruited candidates for this experiment, we used certain criteria derived from a screening session in which 411 university students completed both of the questionnaires described above. The first criterion was whether individuals had primary psychopathy scores on the LSRP 0.5 *SD* above or 0.5 *SD* below the average for the screening session (*M* = 33.01, *SD* = 6.36; thus, 0.5 *SD* = 3.18), which was also used for group allocation. The LSRP can measure the primary and secondary psychopathic traits that correspond to emotional detachment and impulsivity, respectively (see section Measurements for details). We define psychopathy as emotional dysfunction rather than impulsivity because several prior studies have reported defects in emotional responses (Blair et al., 1997; Osumi et al., 2007b), and primary psychopathic traits are theoretically unique to psychopathy (Blair, 2006). Moreover, Blair et al. (2006) revealed that impulsive traits related to secondary psychopathy were unlikely to predict learning performance (however, see Lynam et al., 1999). Therefore, we focused on the difference in primary psychopathy and allowed secondary psychopathy to be matched at the average level in the two groups. The other criterion was used to control for trait anxiety. Learning deficits in psychopathy were often obtained only when individuals had high scores for psychopathic traits and low anxiety (Lykken, 1957; Newman and Schmitt, 1998). Therefore, we refrained from recruiting people with anxiety traits greater than 1 *SD* above the average score of the screening session (*M* = 47.76, *SD* = 8.83). A summary of these personality traits is shown in Table 1.

**TABLE 1:** Means and standard deviations of LSRP and STAI scores by group.

	**Psychopathy**		***t-*value**	***p-*value**
	**High trait scores**	**Low trait scores**		
	**(*n* = 21)**	**(*n* = 22)**		
PP	42.76 (4.62)	25.64 (2.44)	15.29	*p* < 0.001
SP	20.67 (2.33)	19.55 (2.54)	1.51	*p* = 0.140
TA	42.00 (6.32)	44.27 (7.19)	1.10	*p* = 0.278

*PP, primary psychopathy; SP, secondary psychopathy; TA, trait anxiety. Standard deviations are in parentheses.*

#### Measurements

We used the Japanese version of the LSRP (Levenson et al., 1995; Sugiura and Sato, 2005) to assess the participants’ psychopathic tendencies. The LSRP has been examined in terms of its reliability and validity by Lynam et al. (1999) and Osumi et al. (2007a) and has been used by several studies (Osumi et al., 2007b; Kahane et al., 2015; Pletti et al., 2017). The LSRP has two subgroups corresponding to primary psychopathy and secondary psychopathy. Primary psychopathy encompasses callousness and a manipulative attitude toward others (e.g., “People who are stupid enough to get ripped off usually deserve it”), whereas secondary psychopathy involves impulsivity and stimulation-seeking behavior (e.g., “I don’t plan anything very far in advance”). The primary psychopathy subscale consists of 16 items, and the secondary psychopathy subscale includes 10 items. Cronbach’s alpha statistics calculated from the screening session data were 0.790 for primary psychopathy and 0.599 for secondary psychopathy. Each item is rated on a four-point Likert-type scale [from disagree strongly (1) to agree strongly (4)].

The trait anxiety scale from the STAI (Spielberger et al., 1970) is a 20-item self-report questionnaire that measures the level of anxiety in daily life (e.g., “I lack self-confidence”). We used a Japanese version of the STAI, the validity of which was examined by Shimizu and Imae (1981). Cronbach’s alpha for this scale in the screening session was 0.859. Each STAI item is rated on a four-point Likert-type scale [from not at all (1) to very much so (4)].

#### Learning Task

**FIGURE 1:**
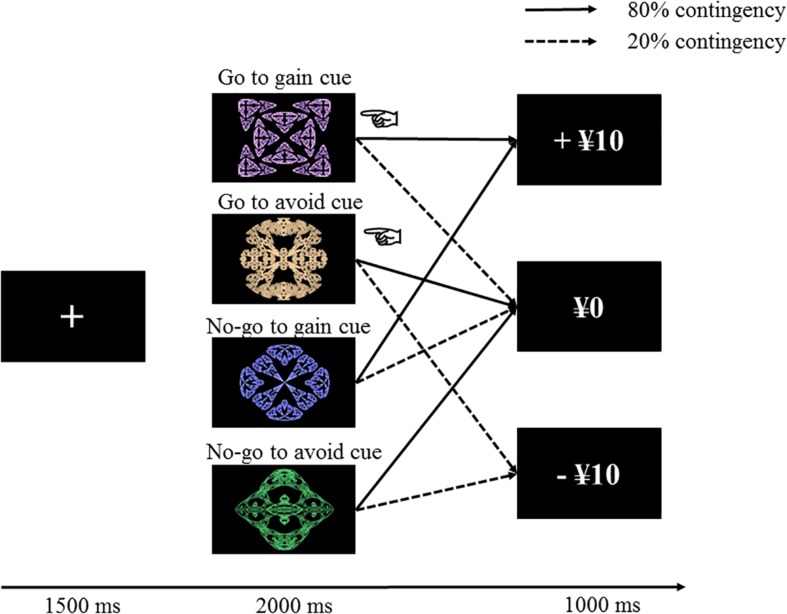
Task structure in this experiment. Correct actions often lead to desirable results (increasing 10 yen in the gain cue and preventing a loss of money in the avoidance cue), whereas incorrect actions generally lead to undesirable consequences (omitting the reward and receiving the punishment). The pointing finger in this figure is depicted as a go action.

The experimental task was a probabilistic go/no-go learning task that is almost identical to that used by Guitart-Masip et al. (2012). The experiment was controlled by PsychoPy v1.80.30 (Peirce, 2009). In this paradigm, participants were required to learn approach or avoidance actions from positive or negative outcomes (Figure 1). At the start of the trial, a fixation cross appeared for 1.5 s on the computer screen. Then, one of four fractal images was presented as a condition stimulus. Participants had to decide whether to press the space key while a fractal image was displayed for 2 s. After a fractal disappeared, feedback of a gain of ¥10, a loss of ¥10, or neither a gain nor a loss was shown, depending on a prior action upon a fractal. The feedback was presented for 1 s, and then the next trial began.

The four fractal images were randomly assigned to four conditions consisting of go to gain, no-go to gain, go to avoid, and no-go to avoid. The reward (+¥10) or no reward (¥0) feedback was shown in the gain trial, while the punishment (−¥10) or no punishment (¥0) result appeared in the loss trial. The outcomes were variable, such that the correct response led to a positive result at 80% but a negative result at 20% and that the incorrect response yielded a negative result at 80% but a positive result at 20%. These four conditions were presented 60 times; thus, the participants completed a total of 240 trials. The trial order was randomized for a block that included the four conditions. Each participant obtained the total amount that they earned at the end of the experiment. The participants were told that the outcomes were probabilistic, and they were required to find the correct response by trial and error to augment the benefit.

#### Reinforcement Learning Models

To assess the characteristics of learning, we applied delta rule RL models, including a combination of several parameters related to this experiment. All models are designed to assign an action value to each action for making decisions. Here, we consider action *a* (go or no-go) in response to stimulus *s* (a fractal image) on trial *t* for the action value *Q*_*t*_(*a*_*t*_, *s*_*t*_). The action value for a chosen action is updated based on the following equation:

(1) $$Q_{t+1}\,\left(a_{t},s_{t}\right)=Q_{t}\,\left(a_{t},s_{t}\right)+\varepsilon \,\delta_{t}$$

(2) $$\delta_{t}=\rho \,r_{t}-Q_{t}\,\left(a_{t},s_{t}\right)$$

where ε is the learning rate governing the degree to which the value is updated. The subjective impact of outcome ρ is a free parameter representing the effect size of the result. The outcome value *r*_*t*_ is 1 for a gain, -1 for a loss, or 0 for no gain or loss in trial *t*. The term ρ*r*_*t*_−*Q*_*t*_(*a*_*t*_, *s*_*t*_) is the PE described as δ_*t*_. Learning proceeds with a decision for each action according to the values, and the probabilities of implementing an action are calculated by the softmax function:

(3) $$p_{t}\,\left(a_{t},s_{t}\right)=\frac{\exp\left(W_{t}\,\left(a_{t},s_{t}\right)\right)}{\sum_{a^{\prime }}\exp\left(W_{t}\,\left(a_{t}^{\prime },s_{t}\right)\right)}$$

where *W*_*t*_(*a*_*t*_, *s*_*t*_) is an action weight corresponding to *Q*_*t*_(*a*_*t*_, *s*_*t*_), except in the models with specific parameters.

We used two additional parameters that were validated in prior studies to explain the go/no-go learning task (Guitart-Masip et al., 2012, 2014). One parameter was called the action bias, which is a tendency to press a button regardless of learning. The bias parameter *b* influences the action value on the weight:

(4) $$W_{t}\,\left(a_{t},s_{t}\right)=\left\{ \begin{array}{cc} Q_{t}\,\left(a_{t},s_{t}\right)+b\; & i\,f\,a_{t}=g\,o\\ Q_{t}\,\left(a_{t},s_{t}\right)\; & e\,l\,s\,e \end{array}\right.$$

The other parameter was the Pavlovian factor, which expresses the effect of a stimulus value. Several studies have reported that stimuli resulting in rewards tend to block action inhibition, while stimuli leading to punishment tend to discourage reactions even though they are not the correct responses (Guitart-Masip et al., 2012, 2014). The action weight is adapted by the Pavlovian factor π as follows:

(5) $$W_{t}\,\left(a_{t},s_{t}\right)=\left\{ \begin{array}{cc} Q_{t}\,\left(a_{t},s_{t}\right)+\pi \,V_{t}\,\left(s_{t}\right)\; & i\,f\,a_{t}=g\,o\\ Q_{t}\,\left(a_{t},s_{t}\right)\; & e\,l\,s\,e \end{array}\right.$$

(6) $$V_{t+1}\,\left(s_{t}\right)=V_{t}+\varepsilon \,\left(\rho \,r_{t}-V_{t}\,\left(s_{t}\right)\right)$$

The stimulus value *V*_*t*_(*s*_*t*_) is updated with the same parameters used by the action value.

We hypothesized that psychopathic traits may be associated with deterioration in the process related to valence; thus, we divided certain parameters to obtain more detail about learning in psychopathy. The learning rate can be separated according to the positive PE (δ > 0) and negative PE (δ < 0). Models that comprise the learning rates for the signed PE allow an asymmetric effect on learning depending on the reception of better or worse results (Cazé and van der Meer, 2013). Furthermore, the learning rate can be divided into both gain and loss domains, indicating that the updating value in the gain domain can differ from that in the loss domain. Four conditions were consistent with the learning rates: a positive PE in a gain (gain: ε_GP_), a negative PE in a gain (absence of reward: ε_GN_), a positive PE in a loss (avoidance of monetary loss: ε_LP_), and a negative PE in a loss (loss: ε_LN_). The subjective impact of outcomes can also differ between a gain (ρ_G_) and a loss (ρ_L_), indicating that the subjective magnitude of positive reinforcers may not be equal to that of negative reinforcers. In sum, we examined 12 parameters and sought the best combination of these parameters.

#### Model Fitting and Comparison

Free parameters were estimated for each participant via a hierarchical type II maximum likelihood estimation, and the procedures were identical to those used in previous studies (Huys et al., 2011; Guitart-Masip et al., 2012 for details). This method assumes that the parameters of each individual are derived from each parameter distribution. We suppose that the population-level distribution for each parameter is a normal distribution. Certain parameters were converted into a suitable form. To perform the estimation, the likelihood was maximized by the expectation-maximization procedure using the Laplace approximation to calculate the posterior probability. We used the Rsolnp package in R^1^ to optimize the likelihood functions.

These models were evaluated with the integrated Bayesian information criterion (iBIC). A smaller iBIC value represents a better model (Huys et al., 2011). Briefly, the iBIC was calculated by using the following procedures: Using the parameter values randomly generated by the population distributions, the likelihood was calculated multiple times (1,000 times here) for each participant data. Next, after dividing the total likelihood of each participant by the number of samples (1,000), these amounts were summed for all participants. Finally, the cost for the number of parameters was added to this value (see Huys et al., 2011 for details). The iBIC values are approximations of the log marginal likelihoods with a penalty for the number of free parameters.

### Results

#### Learning Performance

**FIGURE 2:**
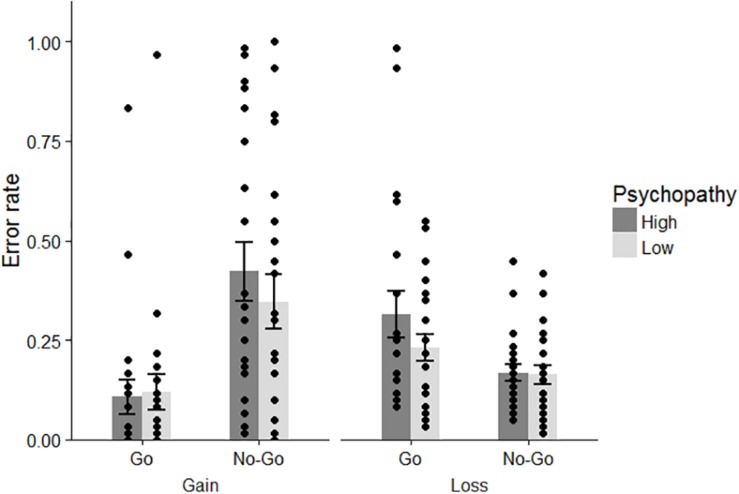
Error rates in each condition for both groups. Dots indicate the data for each participant. Error bars represent standard errors.

For the numbers of errors, we conducted a 2 (psychopathic tendency: high/low) × 2 (correct action: go/no-go) × 2 (domain: gain/loss) repeated-measures ANOVA (Figure 2). This analysis revealed a main effect of action [*F*(1, 41) = 6.315, *p* = 0.016, η*_*p*_*^2^ = 0.134]. Participants made more errors when they needed to suppress a response than when they were required to respond. Consistent with the findings of prior studies, a significant interaction between action and domain was found [*F*(1, 41) = 19.532, *p* < 0.001, η*_*p*_*^2^ = 0.323]. Shaffer’s *post hoc* test indicated that participants were likely to fail to obtain rewards more often by action inhibition (*M* = 0.383, *SD* = 0.329) than by using the go response (*M* = 0.113, *SD* = 0.202; *p* < 0.001), while they showed better performance with the no-go response (*M* = 0.166, *SD* = 0.103) than with the go response (*M* = 0.271, *SD* = 0.221) for avoiding a loss of money (*p* = 0.006). Moreover, the level of error was higher with the go action when participants were engaged in avoiding a monetary loss than when they were engaged in pursuing benefits (*p* = 0.001). In contrast, the number of failures for the no-go response was larger in the gain condition than in the loss condition (*p* < 0.001). For the statistical effects of psychopathic tendency, neither the main effect nor the interactions were significant in learning performance [main effect: *F*(1, 41) = 1.114, *p* = 0.297, η*_*p*_*^2^ = 0.026; psychopathic tendency × action: *F*(1, 41) = 0.004, *p* = 0.949, η_*p*_^2^ = 0.0001; psychopathic tendency × domain: *F*(1, 41) = 0.055, *p* = 0.816, η*_*p*_*^2^ = 0.001; psychopathic tendency × domain × action: *F*(1, 41) = 0.958, *p* = 0.334, η*_*p*_*^2^ = 0.023].

#### Model Selection

**FIGURE 3:**
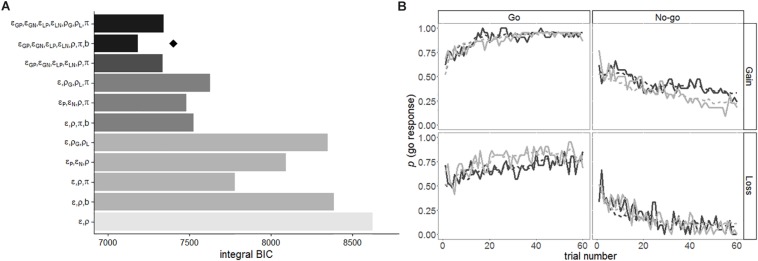
**(A)** Each iBIC value for RL models. ε, learning rate; ρ, subjective impact of outcomes; b, action bias; π, Pavlovian factor. The subscripts represent the following: P, positive PE; N, negative PE; G, gain domain; L, loss domain. The brightness represents the number of parameters (as the number of parameters increases, the bar becomes darker). The diamond shape represents the winning model. **(B)** Average probabilities of choosing a go response in each trial for four conditions and the model predictions. The solid lines indicate the proportions of the go responses in each trial across participants, and the dashed lines show the predictions of the winning model. The black and gray lines represent the high- and low-psychopathy groups, respectively.

Several models that had a specific constellation of free parameters were compared to determine which model yielded the best prediction of the choice data by using the iBIC. Using a stepwise procedure for comparing models, we added one free parameter to a model and accepted the plausible parameter that decreased the iBIC the most at each step. First, as depicted in Figure 3A, the Pavlovian factor π reduced the iBIC of the basic model (one learning rate ε and one subjective impact of outcomes ρ) over the other parameters. The iBIC of the model with π was diminished by separation of the learning rates for positive and negative PEs (ε_P_ and ε_N_). The learning rates that were further divided between gains and losses (ε_GP_, ε_GN_, ε_LP_, and ε_LN_) also decreased the iBIC value. Finally, the action bias parameter *b* reduced the iBIC. The subjective impact of outcomes among gains (ρ_G_) and losses (ρ_L_) did not reduce the iBIC. The winning model included four different learning rates (ε_GP_, ε_GN_, ε_LP_, and ε_LN_) and one subjective impact of outcomes ρ, action bias *b*, and the Pavlovian factor π. Figure 3B shows a prediction of the winning model for the actual choice data.

#### Group Differences of the Parameters

**FIGURE 4:**
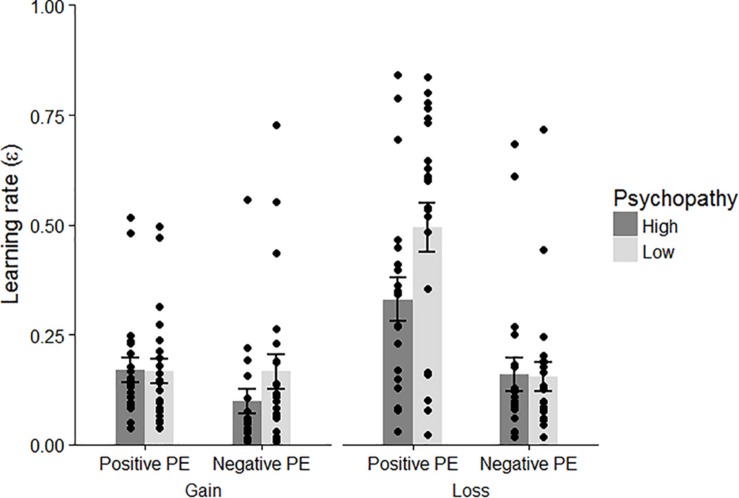
Learning rates for each condition in the psychopathic and non-psychopathic groups. Error bars and dots represent standard errors and individual data, respectively.

We addressed the main question of how learning processes differ between individuals with high- and low-psychopathy traits scores. Using the winning model, we first checked the learning rates. A 2 (psychopathic tendency: high/low) × 2 (type of PE: positive/negative) × 2 (domain: gain/loss) repeated-measures ANOVA was performed (Figure 4). Shaffer’s *post hoc* test was used when a significant interaction was found. The ANOVA was significant for each main effect [psychopathic tendency: *F*(1, 41) = 4.988, *p* = 0.031, η*_*p*_*^2^ = 0.109; type of PE: *F*(1, 41) = 23.401, *p* < 0.001, η*_*p*_*^2^ = 0.363; domain: *F*(1, 41) = 22.378, *p* < 0.001, η*_*p*_*^2^ = 0.353]. The results indicated that participants who scored high on psychopathic tendencies showed less change in their action value than participants who scored low on psychopathy. Furthermore, the learning rates for the loss condition and the positive PE were larger than the learning rates for the gain condition and the negative PE. The interaction between the type of PE and domain was significant [*F*(1, 41) = 17.642, *p* < 0.001, η*_*p*_*^2^ = 0.301], suggesting that participants showed greater change in their action value when they avoided monetary loss than when they experienced monetary gain (*p* < 0.001) and loss (*p* < 0.001). Furthermore, a three-way interaction of psychopathic tendency × domain × type of PE was found [*F*(1, 41) = 5.291, *p* = 0.027, η*_*p*_*^2^ = 0.114]. This analysis showed that compared to the participants with low-psychopathic traits, the high-psychopathic trait participants possessed a lower learning rate for the positive PE in the loss condition (high-psychopathic students: *M* = 0.330, *SD* = 0.228, low-psychopathic students: *M* = 0.494, *SD* = 264; *p* = 0.036), indicating that individuals with high-psychopathic traits showed reduced value updating when avoiding monetary loss. However, both groups exhibited a higher learning rate for avoidance (ε_LP_) than for the other conditions (high-psychopathic students: *M* = 0.170, *SD* = 0.125, *p* = 0.009 for ε_GP_, *M* = 0.160, *SD* = 0.176, *p* = 0.018 for ε_LN_; low-psychopathic students: *M* = 0.169, *SD* = 0.127, *p* < 0.001 for ε_GP_, *M* = 0.154, *SD* = 0.156, *p* < 0.001 for ε_LN_). These results were replicated when using the other models, including four learning rates (i.e., 4 learning rates + one subjective impact of outcomes + the Pavlovian factor or 4 learning rates + the Pavlovian factor + 2 subjective impact of outcomes).

We further examined the relationships between psychopathic traits and other parameters. We performed *t*-tests between the groups for each parameter but found no significant effects [subjective impact ρ : *t*(41) = 0.251, *p* = 0.803, *d* = 0.077; bias: *t*(41) = 0.164, *p* = 0.871, *d* = 0.050; Pavlovian π : *t*(25.272) = 1.161 [with the Welch correction], *p* = 0.257, *d* = 0.360].

## Experiment 2

In Experiment 1, we observed the group difference in learning rates for positive PE in the loss domain (i.e., slow to learn from the experience of avoidance). The extreme groups approach that we used in Experiment 1 can improve the statistical power but contains several problems (Preacher et al., 2005). Furthermore, many studies have investigated the effects of psychopathy as a continuum (Lynam et al., 1999; Newman et al., 2010; Brazil et al., 2013a; Kahane et al., 2015; Aisbitt and Murphy, 2016). We further examined whether psychopathy-related traits are linearly related to the learning parameters.

### Materials and Methods

#### Participants

We recruited 58 undergraduate and graduate students in this experiment. Our sample size was based on previous studies (Brazil et al., 2013a; Aisbitt and Murphy, 2016; Pletti et al., 2017). All participants provided written informed consent and received ¥1,000 for participation. Three participants’ data were excluded from the analysis because of a technical problem during data collection. Therefore, data from 55 participants were used (31 females, mean age = 19.57, *SD* = 1.84).

#### Measurements

We used the same questionnaires as in Experiment 1. Participants filled out the questionnaires after finishing the learning task.

#### Leaning Task

The task was the same as that in Experiment 1.

#### Reinforcement Learning Models

The same RL models and parameters evaluated in Experiment 1 were evaluated.

#### Model Fitting and Comparison

Model fitting and comparison procedures were the same as those used in Experiment 1.

### Results

#### Learning Performance

**FIGURE 5:**
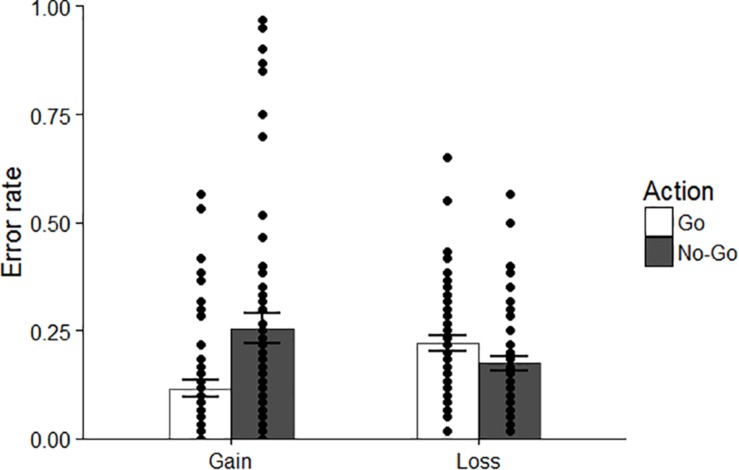
Behavioral performance in Experiment 2. Individual data are displayed by dots. Error bars represent standard errors.

We performed 2 (correct action: go/no-go) × 2 (domain: gain/loss) repeated-measures ANOVA on error rates. Consistent with Experiment 1, the interaction between action and domain was significant [*F*(1, 54) = 22.669, *p* < 0.001, η*_*p*_*^2^ = 0.296; Figure 5]. Using Shaffer’s *post hoc* test, the results for this interaction were identical to those of Experiment 1: compared to rate for the no-go trial, the error rate for the go trial was lower in the gain condition (go to win trials: *M* = 0.113, *SD* = 0.202; no-go to win trials: *M* = 0.383, *SD* = 0.329, *p* = 0.001) but higher in the loss condition (go to avoid trials: *M* = 0.271, *SD* = 0.221, no-go to avoid trials: *M* = 0.166, *SD* = 0.103, *p* = 0.017). In addition, the number of errors with a go response was greater in the loss condition than in the gain condition (*p* < 0.001), whereas the failure made by a no-go response was larger in the gain domain than in the loss domain (*p* = 0.020).

#### Model Selection

**FIGURE 6:**
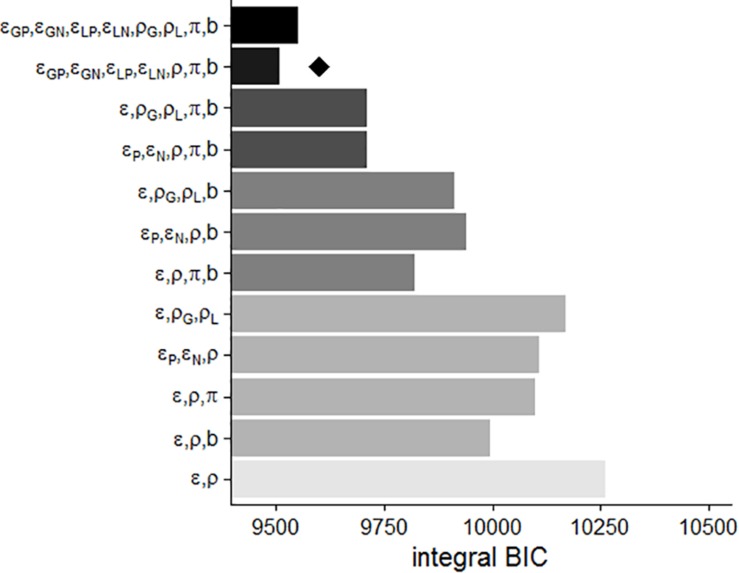
Model comparisons in Experiment 2. ε, learning rate; ρ, subjective impact of outcomes; b, action bias; π, Pavlovian factor. The subscripts represent the following: P, positive PE; N, negative PE; G, gain domain; L, loss domain. The brightness represents the number of parameters. The diamond shape represents the winning model.

As in Experiment 1, we evaluated the RL models by the iBIC values by applying the stepwise method (see Figure 6). The iBIC value of the RL model including a response bias parameter decreased the most compared to that of the basic model. The next free parameter reducing the iBIC value was the Pavlovian factor. Then, the four learning rates decreased the iBIC value. Thereafter, the subjective impacts of outcome divided between the domains did not decrease the iBIC value. The winning model was identical to that of Experiment 1, and we used this model for subsequent analysis.

#### Personality Traits and Learning Parameters

Table 2 shows the descriptive statistics for each personality trait and the correlations with personality traits among the types of learning performances and parameters. In contrast to Experiment 1, only the correlation between primary psychopathy scores and the learning rate for positive PE in the gain domain was significant (*r* = 0.292, *p* = 0.030).

**TABLE 2:** Descriptive statistics and correlations of each personality trait with task performance and learning parameters.

	***M* (*SD*)**	**Error rates**				**Learning parameters**						
		**Go-Gain**	**No-go-Gain**	**Go-Loss**	**No-go-Loss**	**ε_GP_**	**ε_GN_**	**ε_LP_**	**ε_LN_**	**ρ**	**Bias**	**π**
PP	34.11 (5.87)	0.204	–0.023	0.141	0.119	0.292^∗^	–0.060	–0.061	0.123	–0.164	–0.128	–0.155
SP	20.06 (3.47)	–0.008	0.158	0.173	0.172	0.142	–0.034	–0.025	–0.019	–0.028	0.015	–0.052
TA	49.16 (9.26)	0.011	–0.196	–0.153	0.005	0.050	–0.033	–0.083	0.126	0.031	–0.066	0.024

*PP, primary psychopathy; SP, secondary psychopathy; TA, trait anxiety; ε_*GP*_, learning rate for positive PE in gains; ε_*GN*_, learning rate for negative PE in gains; ε_*LP*_, learning rate for positive PE in losses; ε_*LN*_, learning rate for negative PE in losses; ρ, subjective impact of outcomes; bias, response bias; π, Pavlovian factor. ^∗^p < 0.05.*

**FIGURE 7:**
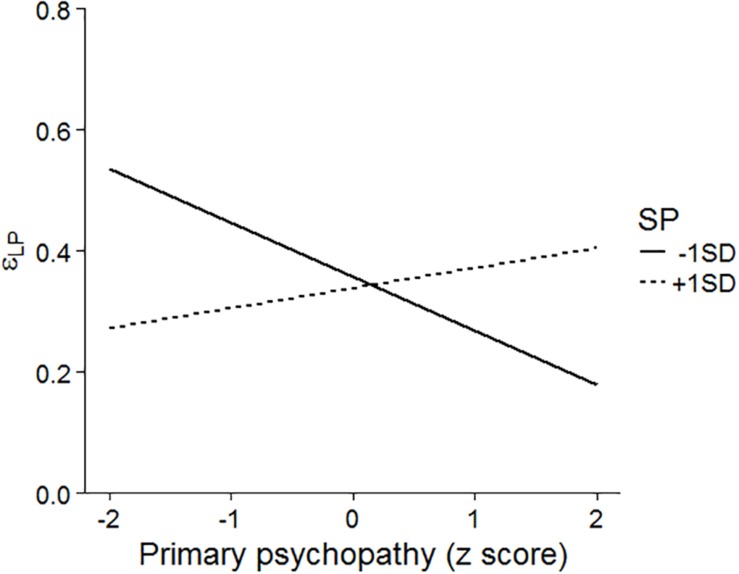
The interaction between primary and secondary psychopathy on the learning rate for positive PE in the loss domain. SP, secondary psychopathy.

We conducted hierarchical regression analyses to investigate further potential relationships between personality traits and learning parameters because some studies reported that learning performance in psychopathy can be modulated by other personality traits, such as anxiety levels (Lykken, 1957; Newman and Schmitt, 1998). In addition, we calculated Bayesian 95% credible intervals (and report highest-density intervals: HDIs) for the coefficients. First, we examined whether the learning parameters were predicted by primary and secondary psychopathic scores on the LSRP. The mean-centered variables were included at step 1, and their interaction was entered at step 2. The results are shown in Table 3. In the learning rate for positive PE in the loss domain, the interaction between primary and secondary psychopathy was significant [Δ*R*^2^ = 0.089, *F*(1, 54) = 5.033, *p* = 0.029; Figure 7]. The simple slope test indicated that, partly consistent with Experiment 1, increased primary psychopathy scores were related to a failure to learn from the avoidance of monetary loss among participants who had low secondary psychopathic traits (β = −0.479, *p* = 0.047, 95% HDI = [−0.984, −0.027]) but not among those who had high secondary psychopathic traits (β = 0.177, *p* = 0.322, 95% HDI = [−0.141, 0.554]).

**TABLE 3:** Results of hierarchical regression analyses for each learning parameter predicted by primary and secondary psychopathy scores.

	**Step 1**			**Step 2**			
	**PP**	**SP**	***R*^2^**	**PP**	**SP**	**PP × SP**	**Δ*R*^2^**
				
	**β**			**β**			
ε_GP_	0.276 [−0.021, 0.553]	0.046 [−0.265, 0.299]	0.087	0.236 [−0.059, 0.538]	0.027 [−0.274, 0.316]	0.143 [−0.137, 0.449]	0.018
ε_GN_	−0.055 [−0.354, 0.231]	−0.015 [−0.310, 0.290]	0.004	−0.037 [−0.354, 0.288]	−0.006 [−0.270, 0.329]	−0.064 [−0.369, 0.263]	0.004
ε_LP_	−0.060 [−0.366, 0.232]	−0.005 [−0.304, 0.284]	0.004	−0.151 [−0.451, 0.142]	−0.048 [−0.348, 0.229]	0.320^∗^ [0.034, 0.625]	0.089^∗^
ε_LN_	0.146 [−0.154, 0.442]	−0.069 [−0.368, 0.232]	0.019	0.172 [−0.146, 0.478]	−0.057 [−0.378, 0.233]	−0.091 [−0.399, 0.209]	0.007
ρ	−0.175 [−0.478, 0.106]	0.032 [−0.278, 0.327]	0.028	−0.141 [−0.485, 0.231]	0.047 [−0.215, 0.374]	−0.118 [−0.417, 0.182]	0.012
bias	−0.152 [−0.434, 0.133]	0.067 [−0.242, 0.334]	0.020	−0.190 [−0.504, 0.101]	0.049 [−0.227, 0.352]	0.136 [−0.184, 0.417]	0.016
π	−0.197 [−0.497, 0.090]	0.120 [−0.165, 0.399]	0.036	−0.169 [−0.470, 0.127]	0.133 [−0.189, 0.411]	−0.097 [−0.369, 0.202]	0.008

*PP, primary psychopathy; SP, secondary psychopathy; β, standardized coefficient; ε_*GP*_, learning rate for positive PE in gains; ε_*GN*_, learning rate for negative PE in gains; ε_*LP*_, learning rate for positive PE in losses; ε_*LN*_, learning rate for negative PE in losses; ρ, subjective impact of outcomes; bias, response bias, π, Pavlovian factor. ^∗^p < 0.05. The 95% highest-density intervals (HDIs) are presented in square brackets.*

**FIGURE 8:**
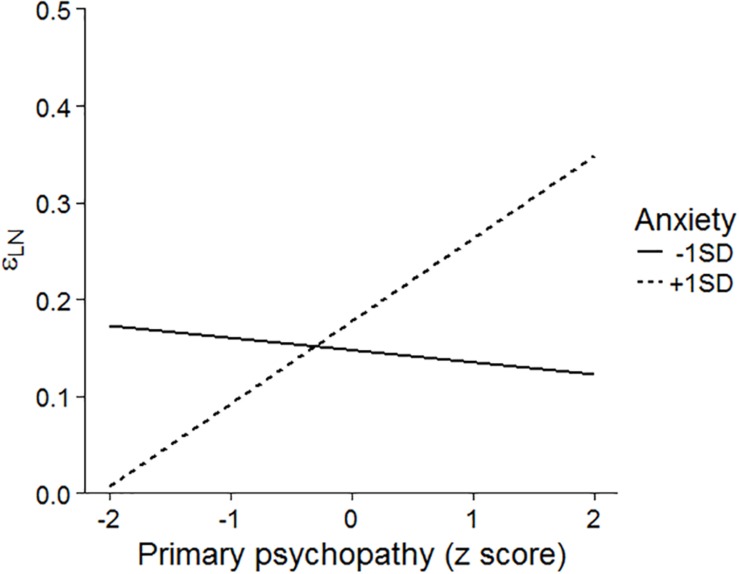
The interaction between primary psychopathy and trait anxiety on the learning rate for negative PE in the loss domain.

Second, the relationships between primary psychopathy and trait anxiety were tested. Table 4 shows the results of hierarchical regression analyses on learning parameters. We found a significant interaction between primary psychopathy and trait anxiety in the learning rate for negative PE in the loss domain (Δ*R*^2^ = 0.088, *F*(1, 54) = 5.062, *p* = 029, β = 0.315, 95% HDI = [0.040, 0.558]; Figure 8). Using the simple slope test, primary psychopathy scores predicted faster learning from negative outcomes when learners had high trait anxiety (β = 0.489, *p* = 0.030, 95% HDI = [0.054, 0.899]) but not when they had low trait anxiety (β = −0.072, *p* = 0.644, 95% HDI = [−0.371, 0.243]). Moreover, trait anxiety was positively correlated with the learning rate among individuals with high primary psychopathic traits (β = 0.367, *p* = 0.045, 95% HDI = [0.013, 0.734]) but not among those with low primary psychopathy (β = −0.194, *p* = 0.311, 95% HDI = [−0.570, 0.191]).

**TABLE 4:** Results of hierarchical regression analyses for each learning parameter predicted by primary psychopathy and trait anxiety scores.

	**Step 1**			**Step 2**			
	**PP**	**TA**	***R*^2^**	**PP**	**TA**	**PP × TA**	**Δ*R*^2^**
				
	**β**			**β**			
ε_GP_	0.295^∗^ [0.008, 0.547]	−0.011 [−0.289, 0.260]	0.086	0.220 [−0.086, 0.492]	0.001 [−0.287, 0.263]	−0.219 [−0.445, 0.064]	0.042
ε_GN_	−0.056 [−0.329, 0.240]	−0.021 [−0.289, 0.289]	0.004	−0.070 [−0.374, 0.221]	−0.019 [−0.325, 0.265]	−0.040 [−0.298, 0.228]	0.001
ε_LP_	−0.046 [−0.337, 0.232]	−0.073 [−0.367, 0.227]	0.009	−0.036 [−0.350, 0.257]	−0.075 [−0.360, 0.224]	0.028 [−0.238, 0.291]	0.001
ε_LN_	0.101 [−0.208, 0.364]	0.105 [−0.197, 0.355]	0.026	0.208 [−0.082, 0.502]	0.086 [−0.170, 0.373]	0.315^∗^ [0.040, 0.558]	0.088^∗^
ρ	−0.178 [−0.438, 0.105]	0.068 [−0.233, 0.350]	0.031	−0.140 [−0.411, 0.158]	0.061 [−0.236, 0.344]	0.111 [−0.149, 0.370]	0.011
bias	−0.120 [−0.396, 0.166]	−0.041 [−0.329, 0.228]	0.018	−0.177 [−0.470, 0.115]	−0.031 [−0.298, 0.256]	−0.169 [−0.417, 0.091]	0.025
π	−0.168 [−0.465, 0.103]	0.059 [−0.213, 0.349]	0.027	−0.161 [−0.455, 0.133]	0.058 [−0.217, 0.354]	0.018 [−0.248, 0.255]	< 0.001

*PP, primary psychopathy; TA, trait anxiety; β, standardized coefficient; ε_*GP*_, learning rate for positive PE in gains; ε_*GN*_, learning rate for negative PE in gains; ε_*LP*_, learning rate for positive PE in losses; ε_*LN*_, learning rate for negative PE in losses; ρ, subjective impact of outcomes; bias, response bias, π, Pavlovian factor. ^∗^p < 0.05. The 95% highest-density intervals (HDIs) are presented in square brackets.*

**FIGURE 9:**
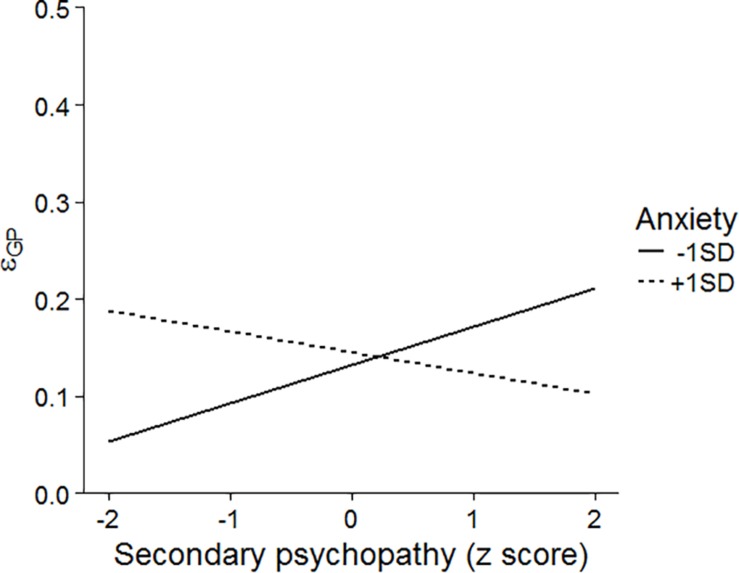
The interaction between secondary psychopathy and trait anxiety on the learning rate for positive PE in the gain domain.

Next, we investigated the effect of interaction of secondary psychopathy and trait anxiety on each of the learning parameters (Table 5). A significant interaction between secondary psychopathy and trait anxiety was observed in the learning rate for positive PE in gains (Δ*R*^2^ = 0.094, *F*(1, 54) = 5.395, *p* = 024, β = −0.312, 95% HDI = [−0.506, −0.033]; Figure 9). The simple slope analysis indicated that secondary psychopathic traits were positively related to faster learning to obtain rewards when participants had low anxiety (β = 0.360, *p* = 0.035, 95% HDI = [0.018, 0.685]) but not when they had high anxiety (β = −0.196, *p* = 0.328, 95% HDI = [−0.584, 0.236]).

**TABLE 5:** Results of hierarchical regression analyses for each learning parameter predicted by secondary psychopathy and trait anxiety scores.

	**Step 1**			**Step 2**			
	**SP**	**TA**	***R*^2^**	**SP**	**TA**	**SP × TA**	**Δ*R*^2^**
				
	**β**			**β**			
ε_GP_	0.138 [−0.158, 0.416]	0.014 [−0.270, 0.294]	0.020	0.082 [−0.197, 0.377]	0.059 [−0.248, 0.333]	−0.312^∗^ [−0.506, −0.033]	0.094^∗^
ε_GN_	−0.027 [−0.322, 0.265]	−0.025 [−0.310, 0.265]	0.002	−0.029 [−0.305, 0.268]	−0.025 [−0.327, 0.264]	−0.006 [−0.265, 0.239]	< 0.001
ε_LP_	−0.004 [−0.286, 0.301]	−0.082 [−0.361, 0.205]	0.007	−0.029 [−0.333, 0.270]	0.062 [−0.375, 0.227]	−0.138 [−0.370, 0.134]	0.018
ε_LN_	0.101 [−0.343, 0.244]	0.105 [−0.147, 0.4144]	0.019	−0.059 [−0.368, 0.206]	0.143 [−0.158, 0.438]	−0.020 [−0.276, 0.226]	< 0.001
ρ	−0.039 [−0.331, 0.264]	0.041 [−0.258, 0.316]	0.002	−0.038 [−0.323, 0.271]	0.040 [−0.251, 0.344]	0.008 [−0.259, 0.259]	< 0.001
bias	0.035 [−0.249, 0.311]	−0.075 [−0.358, 0.211]	0.006	0.002 [−0.272, 0.296]	−0.049 [−0.346, 0.226]	−0.182 [−0.401, 0.094]	0.032
π	0.049 [−0.231, 0.335]	0.011 [−0.277, 0.314]	0.003	0.062 [−0.228, 0.366]	0.001 [−0.312, 0.275]	0.072 [−0.172, 0.330]	0.005

*SP, secondary psychopathy, TA, trait anxiety; β, standardized coefficient; ε_*GP*_, learning rate for positive PE in gains; ε_*GN*_, learning rate for negative PE in gains; ε_*LP*_, learning rate for positive PE in losses; ε_*LN*_, learning rate for negative PE in losses; ρ, subjective impact of outcomes; bias, response bias, π, Pavlovian factor. ^∗^p < 0.05. The 95% highest-density intervals (HDIs) are presented in square brackets.*

Finally, we examined whether the effects of these interactions remained significant when these factors controlled each other. Each mean-centered variable was entered at step 1, and their two-way interactions were included at step 2. Although not all Δ*R*^2^ values were significant (*p*s > 0.157), the effects of the primary psychopathy × secondary psychopathy interaction and the primary psychopathy × anxiety interaction remained significant (primary psychopathy × secondary psychopathy interaction for ε_LP_: β = 0.316, *p* = 0.048, 95% HDI = [0.001, 0.599]; primary psychopathy × anxiety interaction for ε_LN_: β = 0.327, *p* = 0.032, 95% HDI = [0.016, 0.531]). In contrast, the effect of the secondary psychopathy × anxiety interaction was not significant (primary psychopathy × secondary psychopathy interaction for ε_GP_: β = −0.227, *p* = 0.109, 95% HDI = [−0.450, 0.053]).

## Discussion

The goal of the present study is to investigate a computational profile of learning in psychopathy by applying RL models. A deterioration in overall learning performance (i.e., error rate) was not related to psychopathic traits. However, using the RL model-based analysis, learning rates for positive PE in the loss condition tended to be slower for individuals with high-psychopathic traits than for those with low-psychopathic traits, indicating weak updating of the value for avoiding money loss. This relationship was observed in both Experiments 1 and 2. Moreover, in Experiment 2, other learning rates were associated with psychopathic traits. In contrast, we observed no relationships between psychopathy and other learning parameters, such as the subjective impact of outcomes and the Pavlovian factor.

The difference between high and low-psychopathic traits emerged as the learning rate, which controls the speed of updating the value. The common finding in both experiments was the learning deficit in psychopathy under the loss condition, but this finding was related to positive PE, indicating that the result achieved is better (i.e., zero) than the prediction (i.e., negative action value), indicating successful avoidance. This finding indicates that the difference in psychopathic traits is in part related to the process in which learning is accomplished by reducing the error between a prediction and an outcome. In one study of event-related potentials, Bai et al. (2015) used an RL model and revealed that P300 amplitude was correlated with the magnitude of PE. A recent review article for the P300 component in psychopathy showed that psychopathic traits, especially interpersonal-affective traits, were often negatively associated with P300 amplitudes in fear conditioning and picture-affective tasks (Pasion et al., 2018, however, see also Gao and Raine, 2009). If P300 amplitudes can be related to error processing, this finding partially supports our result that individuals with high psychopathy scores have difficulties reducing the error.

In contrast, the result in Experiment 2 suggested that this relation was specific to individuals with low secondary psychopathy scores rather than those with high secondary psychopathy scores. Some studies have reported that impulsive psychopathic traits are related to negative urgency, which is the tendency to act impulsively when one feels negative emotions (Anestis et al., 2009; Weidacker et al., 2017). It can be considered that individuals who score high on secondary psychopathy traits employ avoidance behaviors to reduce their negative emotion; therefore, the learning rate for avoidance did not differ among persons who had high levels of secondary psychopathy. The group difference observed in Experiment 1 may be consistent with this result because the level of secondary psychopathy was controlled among the participants in Experiment 1.

In Experiment 2, we found further relationships between psychopathy and learning rates. These findings, which were not revealed in Experiment 1, might have been due to the methodology, in which the ranges of all personality scores were not constrained. In the gain domain, the learning rate for positive PE was correlated with primary psychopathy and predicted by the interaction between secondary psychopathy and trait anxiety. Both types of psychopathic traits, especially secondary psychopathy, are often associated with abnormal functions and volumes in neural reward systems (Buckholtz et al., 2010; Glenn et al., 2010; Bjork et al., 2012; Korponay et al., 2017), indicating that individuals with psychopathy may show relatively higher reward processing. Our findings related to secondary psychopathy, however, are restricted to low-anxiety individuals. Psychopathic individuals with low anxiety tend to be less influenced by distractor stimuli when engaged in goal-directed behavior (Hiatt et al., 2004; Zeier et al., 2009). It is possible that students with secondary psychopathy and low anxiety saw reward maximization as a high priority, however, these relationships were not significant after controlling for other variables. Nevertheless, the learning processes in the gain domain can provide insights into the different aspects of psychopathy.

In the loss domain, increased primary psychopathy among high-anxiety persons was positively related to the learning rate for negative PE, which determines how fast a person learns from negative outcomes. In addition, students with both high primary psychopathy and trait anxiety scores tended to have greater learning rates for negative outcomes than those with high primary psychopathy but low trait anxiety. Newman et al. (2005) showed that psychopathic persons who have high-anxiety traits were more sensitive to punishments than a control group. These findings may indicate greater sensitivity to punishment. The concept of psychopathy contains certain subtypes, one of which is classified by the level of anxiety (Lykken, 1957; Newman and Schmitt, 1998; Hiatt et al., 2004; Newman et al., 2005; Zeier et al., 2009). The findings in Experiment 2 indicated that anxiety can modulate the relationships between psychopathic traits and the learning process for negative outcomes and that RL models can detect characteristics related to the subtypes of psychopathy (i.e., high- and low-anxiety psychopathy).

In the delta rule RL framework for stochastic avoidance learning, bringing the negative action value to zero seems to be better than decreasing the action value. Indeed, in the two groups, the learning rate for positive PE in losses was larger than that for positive PE in gains (obtaining 10 yen) and negative PE in losses (losing 10 yen). If the learning rate for negative PE in losses is larger than that for positive PE in losses, then the action value of a more avoidable option is likely to become negative. Then, one must experience the consequences of a worse option several times in order to differentiate between the values for the better and worse options. This contrasts with the tendency for a worse option to steer an individual away from his or her preference. Therefore, in the loss condition, the learning rate for positive PE was larger than that for negative PE. However, individuals with high levels of psychopathy tend to require more experiences of avoidance in order to increase the action value to zero from a negative quantity.

In contrast, other learning parameters did not differ on the basis of psychopathy-related scores. The subjective impact of outcomes (as shown in *ρ*), which controls the randomness of choice in RL models, can be interpreted as motivation to seek reward or avoid punishment (Guitart-Masip et al., 2012, 2014; Katahira et al., 2015). Our findings suggest that the motivation of individuals with high-psychopathic tendencies to avoid negative consequences is comparable to that of individuals with low-psychopathic tendencies. In fact, several studies have revealed that individuals with psychopathy often have equal and sometimes more negative ratings than those without psychopathy (Patrick et al., 1993; Baskin-Sommers et al., 2016; Hoppenbrouwers et al., 2016). The findings from these studies imply that individuals with psychopathy maintain subjective negative feelings about negative outcomes. In addition, psychopathy did not relate to either the Pavlovian factor, which is the extent to which action values are influenced by stimulus values, or the action bias, which applies to action tendencies not related to learning. However, these parameters seemed to correspond to behavioral results, such as the main effect of action and the domain × action interaction. These parameters, at least in the present study, can describe general learning functions but not learning in psychopathy.

One of the merits of using RL models is the ability to clarify the learning mechanisms that underlie individual differences (Montague et al., 2012; Huys et al., 2016). Many studies have demonstrated problems of avoidance learning in psychopathy, and several theories have been proposed to account for this deficiency. However, the links between the delta rule RL algorithms and the former theories have remained largely unknown. We draw contrasts between the former theories and our model. First, the low-fear hypothesis accounts for psychopathic behavior by a high reaction threshold for aversive stimuli (Lykken, 1957; Patrick et al., 1993; Hoppenbrouwers et al., 2016). Our results suggest that the learning impairment in psychopathy is caused by poor preventive abilities (i.e., avoidance) but not by the direct drivers of negative emotions, such as updating negative values, which are increased in high-anxiety psychopathy. The low-fear hypothesis can be redefined as the dysfunction of protective abilities in relation to negative stimuli, at least in the present task. Second, in contrast, the response modulation hypothesis emphasizes attentional dysfunction (Hiatt et al., 2004; Zeier et al., 2009; Newman et al., 2010; Newman and Baskin-Sommers, 2016). In Experiment 2, psychopathic traits were positively related to the learning rate for positive PE in gains, indicating that individuals with high psychopathic traits may focus on reward information more than those with low psychopathy. However, in Experiment 1, the group with a psychopathic tendency had a faster learning rate for positive PE in the loss domain compared to the other learning rates; thus, it is still unclear whether the learning rate diminished because the participants in this group paid attention to other information. Nonetheless, the response modulation hypothesis can further our understanding of computational processes in psychopathy. Finally, IES theory, which predicts impairment in forming an association (Blair, 2006), seems to be the most consistent with the current results. However, IES theory largely assumes failure in associations with positive or negative consequences (Blair, 2006). Nevertheless, IES theory is still informative due to its insight into the neural mechanisms of learning in psychopathy. Although many questions remain regarding the relationships between psychopathy and computational mechanisms, these theories provide interpretations of our model, and RL models can represent the theoretical frameworks, at least with respect to learning mechanisms.

Although we showed the computational abnormality of learning in psychopathy, we failed to show a learning performance deficit in the high-psychopathy group. The overall learning performance was consistent with that observed in previous studies (Guitart-Masip et al., 2012, 2014); thus, the learning task in this study was functional. One possible explanation for why we could not observe group differences in learning performance may be related to the demographic characteristics of the participants, who were recruited from a subclinical population. Another explanation may be related to the experimental settings. Many studies use a go/no-go learning task in which the stimulus-outcome association is stable and only two conditions (e.g., go to reward and no-go to avoid) are involved (Newman and Kosson, 1986; Newman and Schmitt, 1998; Lynam et al., 1999). In contrast, our learning task required participants to perform under more complex conditions involving, for example, probabilistic outcomes. Indeed, some studies have shown no differences between individuals with psychopathy and those without psychopathy in probabilistic learning performance during acquisition trials (Budhani et al., 2006; von Borries et al., 2010; Brazil et al., 2013b).

However, our model can predict why performance in probabilistic learning is the same in psychopathy. One possible explanation is that the learning rate in persons with low psychopathy is too high to be associated with good task performance. The greater learning rate for positive PE in the loss condition likely causes lower task performance because learners overestimate the value of worse options when they receive a better result from the worse options. Therefore, the poor task performance caused by a greater learning rate offset a failure of learning induced by a smaller learning rate. Furthermore, individuals with high psychopathic traits had a higher learning rate for positive PE than for negative PE, which seems to be important for success in avoidance learning. This suggests that learning characteristics vary with the level of psychopathic traits even when learning performance is the same. In other words, RL models can be used to uncover hidden factors that have not been revealed by ordinary analyses.

### Future Directions

Our model can provide a new perspective on psychopathic learning, indicating that individuals with high levels of psychopathy tend not to update values when they have avoided a negative result. This model can also be applied to other types of learning. For instance, a considerable number of studies have reported a dysfunction in reversal learning in psychopathy (Newman et al., 1987; Budhani et al., 2006). Our model presumes that individuals with psychopathy are late in learning when contingencies are reversed because they have difficulty rebuilding the association with an avoidable option that had previously led to unpleasant consequences. Moreover, these individuals may struggle to learn which option is avoidable in a condition with stochastic results, and these probabilities are very low (Cazé and van der Meer, 2013). As in the previous examples, the computational model can enable us to consider learning defects in psychopathy.

Future studies should also determine why individuals with psychopathy are likely to neglect information related to avoidance. We speculate that this learning inability in psychopathy may be related to the weak recognition of mental states, that is, beliefs regarding conditions in the external or internal environment. Recently, a new hypothesis regarding OFC function has been proposed, such that it can store mental representations that allow a learner to flexibly transition to a suitable learning and action selection (Wilson et al., 2014). Accumulating evidence has indicated that individuals with psychopathy have weak OFC activation and functions (Birbaumer et al., 2005; Finger et al., 2011; White et al., 2013; Baskin-Sommers et al., 2016). Therefore, individuals with high psychopathy in this experiment may have been late in realizing whether zero represents the avoidance of punishment or the failure to obtain rewards because of confusion about mental states. Future studies need to examine the cause of this impairment.

### Limitations

This study has several limitations. First, as previously mentioned, the participants in this study were recruited from a population of individuals with no criminal history. With regard to grouping, we used the LSRP to measure psychopathy scores, but most studies have used the Psychopathy Checklist-Revised (Hare, 2003), which is often used to assess psychopathic traits. However, several psychopathic characteristics are common in both criminal and non-criminal populations (Lynam et al., 1999; Osumi et al., 2007b; Gao and Raine, 2010; Kahane et al., 2015). We believe that the core components of psychopathy are the same among the whole population and can be captured by all psychopathy measurements. Second, the sample size may be small in each experiment. However, the relationships between psychopathy and learning rates seem robust, at least for the learning rate of avoidance. One problem caused by a small sample size is weak detection of statistical effects (i.e., Type-II error). Type-II error is possible in this study; for example, psychopathic traits might be related to other learning parameters, such as the subjective impact of outcomes. However, our findings may be significant for identifying learning mechanisms in psychopathy, at least in part. Third, we used the paradigm of probabilistic learning, not deterministic learning, because the difficulty level of learning is suitable, whereas deterministic learning appears to be so simple that it may lead to a ceiling effect. The final limitation is related to the types of RL models. While we used RL models that assumed the delta rule, previous research on psychopathy has relied on other learning rules, such as the Bayesian learning rule (Brazil et al., 2013a) or Mackintosh’s associability (Aisbitt and Murphy, 2016). Nonetheless, those previous results are consistent with our findings that individuals with psychopathy have difficulty in forming values.

## Conclusion

In sum, we have provided a computational account of learning deficits with negative consequences in psychopathy, despite recruiting participants from a non-clinical population. We found that individuals who have high levels of psychopathic traits possess abnormal updating processes compared to those of individuals who have low levels of psychopathic traits. In contrast, other learning parameters did not differ on the basis of psychopathy. Our model may at least partially bridge the gap between previous theories and actual learning deficits in psychopathy and expand our understanding of learning impairment.

## Data Availability Statement

The raw data supporting the conclusions of this manuscript will be made available by the corresponding author, without undue reservation, to any qualified researcher.

## Ethics Statement

This study was approved by the Ethics Committee of Nagoya University.

## Author Contributions

TO designed the study, collected the data, and wrote the manuscript. TO and KK analyzed the data. TO, KK, and HO reviewed the manuscript.

## Conflict of Interest

The authors declare that the research was conducted in the absence of any commercial or financial relationships that could be construed as a potential conflict of interest.

## Footnotes

1 https://cran.r-project.org/package=Rsolnp
